# Speech Emotion Recognition Incorporating Relative Difficulty and Labeling Reliability

**DOI:** 10.3390/s24134111

**Published:** 2024-06-25

**Authors:** Youngdo Ahn, Sangwook Han, Seonggyu Lee, Jong Won Shin

**Affiliations:** School of Electrical Engineering and Computer Science, Gwangju Institute of Science and Technology, Buk-gu, Gwangju 61005, Republic of Korea; dori2063@gmail.com (Y.A.); swhan9873@gm.gist.ac.kr (S.H.); lsqjin2022@gm.gist.ac.kr (S.L.)

**Keywords:** speech emotion recognition, out-of-corpus, generalization, relative difficulty, labeling reliability

## Abstract

Emotions in speech are expressed in various ways, and the speech emotion recognition (SER) model may perform poorly on unseen corpora that contain different emotional factors from those expressed in training databases. To construct an SER model robust to unseen corpora, regularization approaches or metric losses have been studied. In this paper, we propose an SER method that incorporates relative difficulty and labeling reliability of each training sample. Inspired by the Proxy-Anchor loss, we propose a novel loss function which gives higher gradients to the samples for which the emotion labels are more difficult to estimate among those in the given minibatch. Since the annotators may label the emotion based on the emotional expression which resides in the conversational context or other modality but is not apparent in the given speech utterance, some of the emotional labels may not be reliable and these unreliable labels may affect the proposed loss function more severely. In this regard, we propose to apply label smoothing for the samples misclassified by a pre-trained SER model. Experimental results showed that the performance of the SER on unseen corpora was improved by adopting the proposed loss function with label smoothing on the misclassified data.

## 1. Introduction

The goal of speech emotion recognition (SER) is to identify emotional states conveyed through spoken utterances. SER can be applied to various areas, including emotional speech synthesis [1], human–computer interaction [2], and mental treatment [3]. Most SER models are constructed by data-driven approaches, demonstrating decent performances, but they may overfit to the training data, resulting in limited performances [4,5,6,7,8,9,10,11]. To increase the size of the training data when the construction of a large-scale emotional speech corpus is difficult, several approaches [7,8,9,10,11,12,13] have used multiple emotional speech corpora in training. Another class of approaches tries to enhance the generalization capability by introducing a variety of regularization approaches and metric losses [7,11,14,15,16,17,18,19,20,21,22,23].

In [17], “soft labels” were proposed to reflect the opinions of all annotators, replacing one-hot vectors, which disregard minor opinions. Label smoothing and unigram smoothing were exploited in [18], where the target label vector was a linear combination between a one-hot vector and a certain class distribution vector, which was a uniform distribution and the class distribution in the training set, respectively. The focal loss [24] was adopted in [19] to give more weights to utterances that were difficult to classify. In [14,20,21,22,23], an unlabeled speech corpus was utilized to construct discriminative latent features with an autoencoder (AE). Pseudo-emotion label (PEL) was introduced in [11], which utilizes an unlabeled speech corpus with the “neutral” labels or all-one label vectors to exploit various expressions in a large database to construct a more robust SER model. Metric learning approaches such as the contrastive loss [25] and triplet loss were utilized in [7,15,16], which learned data-to-data relations by minimizing the distances among embeddings for the samples in the same class and maximizing those for the samples from different classes. In [11], corpus-wise weights (CWW) were introduced to emphasize the samples from the corpora, which were more difficult to classify with the model obtained in the previous epoch of training.

In this paper, to build an SER model that performs well on unseen corpora, we propose an SER method that considers the relative difficulty and labeling reliability (RDLR) of each training sample. Firstly, we propose a novel loss function incorporating the difficulty of each sample inspired by the Proxy-Anchor loss [26], which assigns higher gradients to the harder examples within the given minibatch. As the CWW [11] improved the generalization by paying more attention to the corpora, which make it difficult to classify the emotional state, it may be beneficial to weigh each utterance differently even within the same corpus according to the difficulty of the emotion classification for the given sentence. In addition, we evaluate the reliability of the emotion label for each sample and refine the labels which are considered to be unreliable. Since most of the emotional speech datasets are annotated based on the multimodal data in conversational situations, the emotion labels may be based on conversational context [27] or modalities other than speech (Related examples are available at https://dori2063.github.io/RDLR/ (accessed on 23 June 2024)). Thus, some of the emotion labels cannot be reliably estimated from the current utterance of speech, which may degrade the performance of the SER. To mitigate this issue, we propose to apply label smoothing for the samples misclassified by a pre-trained SER model assuming that the labels for them are unreliable. Experimental results showed that the performance of the SER on unseen corpora was improved by adopting the proposed loss function considering the relative difficulty of the emotion classification among samples and the reliability of the emotion labels.

## 2. Methods

### 2.1. Relative Difficulty-Aware Loss

We propose a relative difficulty-aware (RD) loss inspired by the Proxy-Anchor loss [26], which is one of the proxy-based metric losses [26,28]. A proxy is a representation for each class in the training data, which is learned as a part of the network parameters. Proxy-based metric losses help to learn discriminative embeddings by comparing proxies and data, in contrast to the previous data-based metric losses such as triplet loss which compare data. The first proxy-based loss was the Proxy-Neighborhood Component Analysis (NCA) loss [28], which is defined as

(1) $$L_{\mathrm{NCA}}\left(Z,P\right)=\sum_{z\in Z}-\log\left(\frac{e^{s(z,p^{+})}}{\sum_{p^{-}\in P^{-}}e^{s(z,p^{-})}}\right)$$

where *Z* is a set of embedding vectors in a minibatch, *P* indicates the set of all proxies, $p^{+}$ is a positive proxy corresponding to the class of *z*, $P^{-}$ is the set of negative proxies which includes all proxies in *P* except $p^{+}$, and s(·,·) denotes the cosine similarity between two vectors. The gradient of the Proxy-NCA loss with respect to s(z,p) is given by

(2) $$\frac{\partial L_{\mathrm{NCA}}\left(Z,P\right)}{\partial s(z,p)}=\left\{\begin{array}{cc} -1, & \mathrm{if}\;p=p^{+},\\ \frac{e^{s(z,p)}}{\sum_{p^{-}\in P^{-}}e^{s(z,p^{-})}}, & \mathrm{otherwise}. \end{array}\right.$$

**Figure 1:**
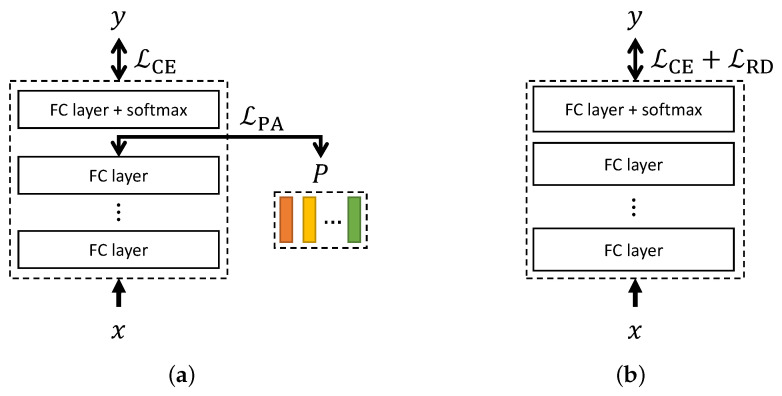
Block diagrams of speech emotion recognition models incorporating (**a**) the Proxy-Anchor loss $L_{\mathrm{PA}}$ and (**b**) the proposed relative difficulty-aware loss $L_{\mathrm{RD}}$. $L_{\mathrm{CE}}$ denotes the cross-entropy loss and *P* represents the set of proxies. The models consist of fully connected (FC) layers. *x* and *y* represent the input feature and the target label.

It shows that minimizing the loss encourages *z* and $p^{+}$ to be close to each other by a constant, and *z* and $p^{-}$ to be far away by their relative similarities. In [26], the Proxy-Anchor loss is proposed to consider both proxy-to-data and data-to-data relations in the evaluation of the gradient. The Proxy-Anchor loss is given by

(3) $$\begin{array}{cc} L_{\mathrm{PA}}\left(Z,P\right) & =\frac{1}{|P^{+}|}\sum_{p\in P^{+}}\log\left(1+\sum_{z\in Z_{p}^{+}}e^{-\alpha (s(z,p)-\delta )}\right)\\ \; & +\frac{1}{\left|P\right|}\sum_{p\in P}\log\left(1+\sum_{z\in Z_{p}^{-}}e^{\alpha \left(s(z,p)+\delta \right)}\right) \end{array}$$

where $P^{+}$ denotes the set of proxies corresponding to the classes into which one or more training samples in the given minibatch fall. Given the proxy *p*, $Z_{p}^{+}$ is the set of embedding vectors in *Z* which belongs to the class *p* represents, and $Z_{p}^{-}=Z-Z_{p}^{+}$. δ is a margin and α is a scaling factor. It can be seen in (3) that $L_{\mathrm{PA}}$ becomes lower when s(z,p) for the embedding vectors $z\in Z_{p}^{+}$ increases and s(z,p) for $z\in Z_{p}^{-}$ decreases for each *p*. It is verified by evaluating the gradient of the Proxy-Anchor loss with respect to s(z,p), which becomes

(4) $$\frac{\partial L_{\mathrm{PA}}\left(Z,P\right)}{\partial s(z,p)}=\left\{\begin{array}{cc} \frac{1}{|P^{+}|}\frac{-\alpha h_{p}^{+}\left(z\right)}{1+\sum_{z^{\prime }\in Z_{p}^{+}}h_{p}^{+}\left(z^{\prime }\right)}, & \forall z\in Z_{p}^{+},\\ \frac{1}{\left|P\right|}\frac{\alpha h_{p}^{-}\left(z\right)}{1+\sum_{z^{\prime }\in Z_{p}^{-}}h_{p}^{-}\left(z^{\prime }\right)}, & \forall z\in Z_{p}^{-}, \end{array}\right.$$

where $h_{p}^{+}\left(z\right)=e^{-\alpha (s(z,p)-\delta )}$ and $h_{p}^{-}\left(z\right)=e^{\alpha (s(z,p)+\delta )}$, which can be thought as a measure of how complex the correct classification of the embedding vector *z* is. We can see that the Proxy-Anchor loss considers proxy-to-data relations via s(z,p), while it also incorporates data-to-data relations as the right-hand side of (4) is $-h_{p}^{+}$ or $h_{p}^{-}$ normalized by those for other embeddings. In [11], the introduction of the CWW to emphasize the samples from the corpora is more difficult than to classify the improved generalization to the unseen corpora when the model is trained with multiple training corpora. For an emotion classifier *F* with a softmax function, the one-hot class label vector $y_{i}$, and the input feature $x_{i}$, the classification loss function with the CWW for a minibatch $\left(X,Y\right)={\left\{x_{i},y_{i}\right\}}_{i=1}^{M}$ is given by

(5) $$L_{\mathrm{CWW}}\left(X,Y,F\right)=-\frac{1}{M}\sum_{i=1}^{M}w_{i}\beta_{i}y_{i}\cdot \log F(x_{i})$$

in which *M* is the number of samples in a minibatch, · represents an inner product, $\beta_{i}$ is the class weight to relieve the bias caused by class-imbalanced training data, and $w_{i}$ is the CWW depending on the emotion classification difficulty of the training database which the *i*-th sample comes from. $w_{i}$’s are initialized to 1 and updated for each training epoch as follows:

(6) $$w_{i}=\frac{{\left(1-U_{d_{i}}\right)}^{\lambda_{W}}}{\frac{1}{D}\sum_{d=1}^{D}{\left(1-U_{d_{i}}\right)}^{\lambda_{W}}}$$

where *D* is the number of training corpora, $d_{i}$ is the corpus index for the *i*-th sample, $U_{d_{i}}$ is the unweighted accuracy (UA) [11] for the corpus $d_{i}$, and $\lambda_{W}$ is a control parameter. Although the CWW only considers relative difficulty of each training corpus to enhance the generalization of the SER, we may expect that considering the relative difficulty of classification among samples within a minibatch would also be helpful as in the Proxy-Anchor loss. We may consider to adopt the Proxy-Anchor loss to SER by applying the Proxy-Anchor loss to the embeddings at the middle of the SER model along with the conventional cross-entropy (CE) loss for the final output, as shown in Figure 1a.

However, the SER deals with known emotional classes and thus the Proxy-Anchor loss to cope with unseen classes may not be the most effective way as it does not evaluate the difficulty of classification for the final output.

To consider the relative difficulty of each sample within the minibatch for emotion classification, we propose a loss function for the last layer of the emotion classifier *F* with a softmax function in which the one-hot class label vector $y_{i}$ for the input $x_{i}$ is used instead of the proxy, as shown in Figure 1b. In virtue of the softmax function, the cosine similarity between $F(x_{i})$ and other one-hot vectors corresponding to the second term in (3) would not be crucial. The proposed RD loss is given by the following simple equation:

(7) $$L_{\mathrm{RD}}\left(X,Y,F\right)=\log\left(\frac{1}{M}\sum_{i=1}^{M}e^{-\alpha (s\left(F\left(x_{i}\right),y_{i}\right)-\delta )}\right),$$

The gradient of the RD loss with respect to $s(F\left(x_{i}\right),y_{i})$ becomes

(8) $$\frac{\partial L_{\mathrm{RD}}\left(X,Y,F\right)}{\partial s(F\left(x_{i}\right),y_{i})}=\frac{-\alpha e^{-\alpha (s\left(F\left(x_{i}\right),y_{i}\right)-\delta )}}{\sum_{j=1}^{M}e^{-\alpha (s\left(F\left(x_{j}\right),y_{j}\right)-\delta )}},$$

which has a higher value for the samples where it is more difficult to predict the emotion labels within a minibatch.

### 2.2. Training Target Considering Labeling Reliability

**Figure 2:**
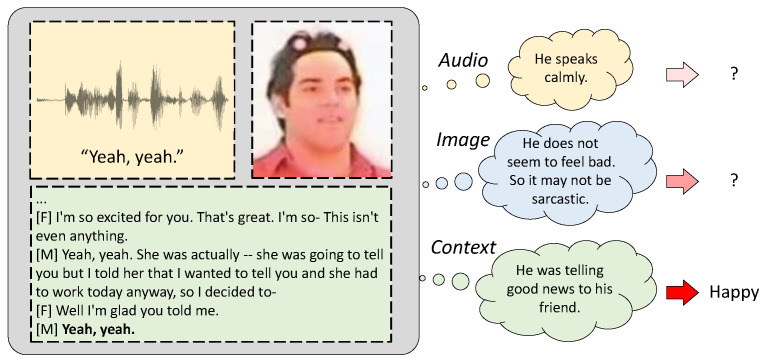
An example in the IEMOCAP dataset for which the emotion is not clear in the current speech utterance but can be inferred by the conversational context.

Speech samples to train an SER model are mostly from emotional datasets for which the annotations were made based on multimodal data in conversational situations. Therefore, some of the emotional labels were decided based on non-speech modalities or conversational contexts, although the labeled emotions were not evident in the speech signals. Figure 2 illustrates an example in the IEMOCAP dataset [29], for which the emotion was not clearly expressed in the given sentence of speech. In this example, the emotion becomes clear only when the conversational context is given. More examples can be found in our demo page^1^. The samples with this type of improper labels may be more problematic for the proposed RD loss, because the RD loss would identify those samples as difficult ones to classify and emphasize them, although they should be treated as mislabeled data. To mitigate this issue, we propose to construct a training target vector considering labeling reliability. In [18], the linear combination between the one-hot label vector and the all-one vector was used as a training target, which is called the label smoothing (LS). The target vector with the LS is given by

(9) $${\bar{y}}_{i}=\left(1-\gamma \right)\times y_{i}+\frac{\gamma }{C}$$

where *C* is the number of classes and γ is a smoothing parameter. We may interpret that the LS assumes that all the annotations are not reliable to an extent. In the proposed method, we apply the LS to the unreliable one-hot label vectors or label-smoothed vectors, which are determined by a pre-trained SER model. The utterances in the training data which are not correctly classified by a pre-trained model are regarded as those with unreliable labels and additional LS is applied as follows:

(10) $${\bar{y}}_{i}=\left\{\begin{array}{cc} \left(1-\gamma \right)\times y_{i}+\frac{\gamma }{C}, & \mathrm{if}\;\mathrm{argmax}\left({\hat{y}}_{i}\right)\neq \mathrm{argmax}\left(y_{i}\right),\\ y_{i}, & \mathrm{otherwise}, \end{array}\right.$$

where ${\bar{y}}_{i}$ is the training target vector considering labeling reliability of each sample and ${\hat{y}}_{i}$ is the prediction output of the pre-trained SER model. We used the same structure with the proposed system without considering labeling reliability (LR) as the pre-trained model.

### 2.3. Speech Emotion Recognition Incorporating Relative Difficulty and Labeling Reliability

The final loss function of the proposed method incorporating relative difficulty and labeling reliability (RDLR) includes the RD loss in (7) along with the CE loss with CWW, $L_{\mathrm{CWW}}$, the autoencoder-based reconstruction loss, $L_{\mathrm{AE}}$, and the CE loss on a non-emotional speech corpus with pseudo-emotion labels (PELs), $L_{\mathrm{PEL}}$, following the loss in [11], i.e.,

(11) $$\begin{array}{c} L_{\mathrm{proposed}}\left(X,\bar{Y},F,H\right)=L_{\mathrm{CWW}}\left(X,\bar{Y},F\right)+\lambda_{\mathrm{RD}}L_{\mathrm{RD}}\left(X,\bar{Y},F\right)\\ +\lambda_{\mathrm{AE}}L_{\mathrm{AE}}\left(X,F^{\prime },H\right)+\lambda_{\mathrm{PEL}}L_{\mathrm{PEL}}\left(X_{\mathrm{unlabeled}},F\right). \end{array}$$

In (11), $\bar{Y}={\left\{{\bar{y}}_{i}\right\}}_{i=1}^{M}$ is the emotion label set obtained by (10), and λ’s are parameters to control relative weights for loss functions. $L_{\mathrm{AE}}$ is given by

(12) $$L_{\mathrm{AE}}\left(X,F^{\prime },H\right)=-\frac{1}{M}\sum_{i=1}^{M}{∥x_{i}-H\left(F^{\prime }\left(x_{i}\right)\right)∥}_{2}^{2}$$

where $F^{\prime }$ is the encoder which has the same structure with the first few layers of *F*, and *H* is the decoder. $L_{\mathrm{PEL}}$ is represented as

(13) $$L_{\mathrm{PEL}}\left(X_{\mathrm{unlabeled}},F\right)=-\frac{1}{M^{\prime }}\sum_{i=1}^{M^{\prime }}{\tilde{y}}_{i}\cdot \log F(x_{i}^{\mathrm{unlabeled}})$$

where $x_{i}^{\mathrm{unlabeled}}$ and ${\tilde{y}}_{i}$ are the input feature and PEL for the *i*-th sample of the unlabeled speech corpus, and $M^{\prime }$ is the number of minibatch for unlabeled speech samples. $X_{\mathrm{unlabeled}}$ is the input feature set for the minibatch. All-one vectors are used as the PELs.

## 3. Experiments

### 3.1. Experimental Design

In our experiments, we employed 4 different emotional speech corpora in English: CREMA-D (CRE) [30], IEMOCAP (IEM) [29], MSP-IMPROV (IMP) [31], and MSP-Podcast (POD) [32]. We considered 4 categorical emotions that were typically used in SER [6,7,8,10,11] including neutral, happy, sad, and angry within each corpus. The specifications on the corpora are summarized in Table 1. CRE is an audiovisual corpus for which 91 professional actors expressed emotions with predefined 12 sentences [30]. IEM is an audiovisual dyadic conversational corpus which consists of 5 sessions. In each session, one actor and actress conversed on a pre-determined topic [29]. To balance the class distribution in IEM, we merged the excitement class into the happy class. IMP is a multimodal emotional corpus spoken by 12 actors engaged in paired interactions across 6 sessions similar to IEM. IMP also included natural speech recorded in the conversations while the actors were not acting [31]. POD is sourced from podcast recordings and encompasses diverse lexical information [32]. We used the released version 1.8, which consists of 28,602, 4772, and 12,787 samples for the train, validation, and test sets, respectively. In POD, the number of labeled speakers is 1285 but also contains samples without speaker labels.

To evaluate the performance of SER models for unseen corpora, we carried out leave-one-corpus-out experiments in which three corpora were utilized for training and validation, and the remaining corpus was used for the test. CRE, IEM, and IMP were divided into 58 and 33 speakers, 4 and 1 sessions, and 5 and 1 sessions for training and validation, respectively. For POD, we used the predefined training and validation sets. We also presented the performances of within-single-corpus SER. As for the within-single-corpus SER, we randomly selected 10, 9, and 72 speakers for the test, validation, and training for CRE, respectively. For IEM, 8 speakers in 4 sessions were used for training; 1 speaker in the last session was used for the validation, and the last speaker was used for the test. For IMP, 6 sessions were divided in a similar way to IEM. For POD, we used the provided partition of data.

We trained the same SER model using soft label [17], label smoothing and unigram smoothing [18], focal loss [19], autoencoder-based unsupervised learning (AE) [20], contrastive loss, Proxy-Anchor loss [26], CWW and PEL [11], and the proposed RD loss and labeling reliability (LR), respectively. For the AE and PEL, we used the Librispeech 100 h [33], which contains 28,539 utterances of audiobooks in English. The contrastive and Proxy-Anchor losses were calculated for the last embedding features of the emotion classifier *F*. In addition, we conducted self-knowledge distillation (Self-KD) [34,35] in order to demonstrate that the performance improvement of the LR did not come from the utilization of a pre-trained SER model. For the Self-KD, we used the prediction output of the pre-trained SER model as the target label and trained a new SER model with the CE loss.

### 3.2. Input Features and Model Configuration

**Figure 3:**
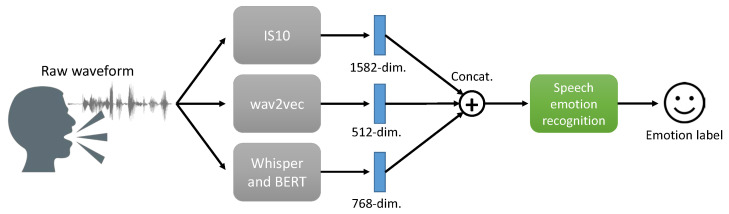
The procedure of input feature processing for speech emotion recognition with IS10, wav2vec, and BERT feature set.

As the input feature *x*, we used the 1582 dimensional IS10 [36] utterance-level feature set with and without wav2vec (W2V) representation [37] and the text-based feature obtained using BERT [38]. We used the openSMILE toolkit [39] to extract the IS10 feature set which was calculated by 21 statistical functionals for 38 low-level descriptors. We extracted the W2V features from the context network of wav2vec and mean pooled to obtain a 512-dimensional utterance-level feature set. As for the text-based features, we used a speech recognition model, Whisper (we used medium.en at https://github.com/openai/whisper, (accessed on 23 June 2024)) [40] to extract text information and then transformed them into 768-dimensional BERT (we used BERT-based-uncased at https://github.com/google-research/bert, (accessed on 23 June 2024)) features. The features were concatenated with the IS10 feature set to form the input *x* when they were used together as shown in Figure 3. The input feature *x* is z-normalized with the means and variances of the training data.

The emotion classifier *F* comprises five fully connected layers with 1024, 1024, 512, 512, and 4 units, where the activation function for the last layer was softmax. The activation functions for all other layers were ReLU and the dropout rate was 0.5. For AE, we used the first two fully connected layers of *F* as the encoder $F^{\prime }$, while the decoder *H* consisted of fully connected layers with 1024, 1024, and input dimensional units. For LS, we used 0.1 as the label smoothing parameter. For RD loss, we fixed α and δ as 1 and 0, respectively, although the performance was not sensitive to these parameters in the experiments. $\lambda_{\mathrm{PEL}}$ and $M^{\prime }$ was set to 0.0001 and 32. The minibatch size *M*, γ, $\lambda_{W}$, $\lambda_{\mathrm{RD}}$, and $\lambda_{\mathrm{AE}}$ were selected from [1024,2048,4096], [0.2,0.3,0.4,0.5,0.6,0.7,0.8], [1,2,3,4], [0.1,0.01,0.001], and [0.5,0.1,0.01,0.001], respectively, and the best results for the unseen corpora are shown in the table. The hyper-parameters for the compared methods were also tuned to achieve the best performances. The code for the proposed method is available (https://github.com/dori2063/RDLR (accessed on 23 June 2024)).

We used Pytorch 1.13 [41] to train the models and Adam optimizer [42] with a learning rate of 0.0002. We measured our SER performance with the UA, which is the average of the accuracies for individual emotional classes. The UA for the validation set was used for the stopping criterion. An early stopping strategy with a patience of 5 was employed using the average UA for training corpora. Also, we used a learning rate scheduler which reduced the learning rate by multiplying 0.1 after 2 patience. We experimented with 5 random seed initializations and reported the averaged results.

## 4. Results

Table 2 summarizes the UAs for the within-corpus and out-of-corpus SER. The test corpus is shown on the top row, and the remaining three corpora were used for training. The average UA for the four experiments for each corpus is shown in the rightmost column. For each corpus and feature configuration of the out-of-corpus SER, the best performance of each target corpus is marked in boldface. The performance for the within-single-corpus SER is also shown in the table, which provides the upper bound of the performance of the out-of-corpus SER and also implies the difficulty of the SER for each corpus. The highest and lowest UA for the within-single-corpus SER were observed in CRE and POD, which collected emotional speech in recording with restricted verbal contents and real conversation, respectively.

In out-of-corpus SER experiments, most of the compared methods demonstrated better performance than the basic SER system with the CE loss. Among the combinations of the previously proposed methods, CWW+† showed the best average UA of 46.3%. When we additionally apply the Proxy-Anchor loss for the last embedding features of *F* on top of CWW+†, the performance slightly increased to 46.6%. The Self-KD did not show a good performance, possibly because the pre-trained SER model did not provide target labels which are good enough to guide the SER model better than the one-hot labels. The RD loss by itself represented an average UA of 45.5%, while it showed 47.3% when combined with CWW+†. The LR resulted in 45.4% of average UA, which is higher than that for the LS or unigram smoothing, implying that the selective application of the label smoothing was effective, and demonstrated 47.3% of the average UA when used with CWW+†. When the LS was replaced by the LR in the CWW+†, which showed the best performance among combinations of the published methods, the UA was improved from 46.3% to 47.3%. The LR improved the performance of the RD+CWW+† when it replaced the LS by 1.0%p. These results show that the label smoothing on the misclassified data could provide the robustness to the ambiguous emotional cues in speech data demonstrated in Figure 2. We could not find further improvement when both the LS and LR were used. When both the RD loss and the target vector considering LR (RDLR) were incorporated with CWW+†, which is the proposed loss function shown in (11), the average UA was 48.4%, which was the best among all compared methods using the IS10 input feature vectors.

Table 3 shows the results when the W2V features were used along with the IS10 feature set and the BERT features were additionally used with the IS10 and W2V features, respectively. The highest average UA was observed when IS10, W2V, and BERT embeddings were used altogether as the input feature. We could observe that the performances were improved by incorporating additional input feature sets including W2V and BERT, and the proposed method showed the best performance for each input feature set. The best performance with the proposed method using IS10, W2V, and BERT features was 54.7%.

It is noted that the proposed method does not affect the computational complexity of the SER in the inference phase, as it only modifies the loss function and the training target. The back-propagation with the RD loss takes additional time in training, although the additional computation in the training phase is smaller than that for the Proxy-Anchor loss which computes the cosine similarities between higher-dimensional vectors. The LR does not introduce additional computation once the smoothed labels are prepared.

## 5. Conclusions

In this paper, we propose a loss function for speech emotion recognition by incorporating the relative difficulty of SER for each training utterance with the training target and considering labeling reliability. The RD loss is designed so that the gradient becomes higher for the samples harder to classify within a given minibatch. In addition, we used soft labels as training target vectors by applying label smoothing for the data misclassified by a pre-trained SER model. Our experimental results demonstrated that RDLR improved the SER performance on unseen corpora compared to previous methods. The proposed method may enhance the performance of the cross-language SER if the training corpora also include diverse languages, but the current model trained with multiple English corpora would not enhance the generalization to another language because the ways to express and perceive emotions are different in different cultures [43].

## Figures and Tables

**Table 1:** Numbers of utterances in each emotional class and numbers of speakers in the corpora used in the experiments.

Corpus	#Speakers	Neutral	Happy	Sad	Angry
CRE [30]	91	1087	1271	1270	1271
IEM [29]	10	1708	1636	1084	1103
IMP [31]	12	3477	2644	885	792
POD [32]	1285+	26,009	14,285	2649	3218

**Table 2:** Unweighted accuracies (%) of speech emotion recognition with IS10 input features for the test corpus on top. Except for the “within-single-corpus (CE)”, the model was trained with the remaining three corpora. RDLR stands for the combination of relative difficulty and labeling reliability that is proposed in this study. † represents the combination of AE, LS, and PEL.

Method	CRE	IEM	IMP	POD	Avg
Within-single-corpus (CE)	66.0	60.1	49.5	46.0	55.4
Out-of-corpus (CE)	51.6	50.1	38.9	31.9	43.1
Soft label [17]	52.7	50.2	40.2	31.6	43.7
Label smoothing (LS) [18]	53.5	51.5	39.4	32.4	44.2
Unigram smoothing [18]	55.0	52.6	39.0	32.7	44.8
Focal loss [19]	51.4	49.6	40.5	32.9	43.6
AE [20]	55.2	48.9	42.8	31.3	44.6
CWW [11]	53.8	52.3	42.7	33.1	45.5
Contrastive loss [25]	52.2	51.4	42.8	32.7	44.8
Proxy-Anchor [26]	52.5	51.5	43.2	33.4	44.9
CWW + PEL [11]	53.5	51.2	40.7	38.7	46.0
AE + LS + PEL (†)	53.1	52.5	42.5	36.1	46.0
CWW + †	53.8	53.4	42.0	36.1	46.3
Proxy-Anchor + †	53.1	52.8	43.8	35.2	46.2
CWW + Proxy-Anchor+ †	54.0	53.1	43.4	35.8	46.6
Self-KD	30.1	33.8	35.2	26.3	31.4
Relative difficulty (RD)	53.3	53.0	43.6	32.2	45.5
CWW + RD	53.5	53.6	**45.2**	32.8	46.3
Labeling Reliability (LR)	53.1	52.9	42.9	32.7	45.4
LS + LR	53.1	53.0	42.9	32.6	45.4
RD + CWW + †	54.0	**54.1**	43.9	37.2	47.3
LR + CWW + AE + PEL	54.3	53.6	43.9	37.4	47.3
LR + CWW + †	54.2	53.6	43.9	37.4	47.3
RD + LR (RDLR)+ †	54.2	54.0	**45.2**	37.7	47.7
RDLR + CWW + AE + PEL	56.3	54.1	44.2	38.7	48.3
RDLR + CWW + † (proposed)	**56.4**	**54.1**	44.3	**38.8**	**48.4**

For each corpus and feature configuration of the out-of-corpus SER, the best performance of each target corpus is marked in boldface.

**Table 3:** Unweighted accuracies (%) for the test corpus on top. Except for the “Within-single-corpus (CE)”, the model was trained with the remaining three corpora. † represents the combination of AE, LS, and PEL. # + X represents X is used as input features in addition to the IS10.

Method	CRE	IEM	IMP	POD	Avg
Within-single-corpus (CE)	66.0	60.1	49.5	46.0	55.4
# + W2V	70.9	62.6	53.1	49.0	58.9
# + W2V and BERT	71.3	68.7	60.3	56.2	64.1
RDLR + CWW + † (proposed)	56.4	54.1	44.3	38.8	48.4
CE # + W2V	52.6	55.9	48.6	36.5	48.4
AE + LS + PEL (†)	53.5	56.2	48.9	39.1	49.6
CWW + †	53.7	56.8	49.9	39.3	49.9
Proxy-Anchor+ †	53.6	56.9	50.5	39.1	50.0
CWW + Proxy-Anchor + †	53.7	56.9	50.6	39.5	50.2
RD + CWW + †	53.4	57.1	50.6	41.3	50.6
LR + CWW + †	53.8	57.1	50.0	40.5	50.4
RDLR + †	**55.5**	57.1	49.8	40.7	50.8
RDLR + CWW + † (proposed)	**55.5**	**57.4**	**50.8**	**41.7**	**51.4**
CE # + W2V and BERT	52.0	60.4	53.1	42.9	52.1
AE + LS + PEL (†)	52.6	61.3	53.6	44.1	52.9
CWW + †	54.9	60.7	52.8	44.2	53.2
Proxy-Anchor + †	53.5	60.8	54.0	44.0	53.1
CWW + Proxy-Anchor + †	54.1	60.9	54.2	44.3	53.4
RD + CWW + †	53.2	61.3	54.6	45.9	53.8
LR + CWW + †	53.4	61.7	**54.8**	**46.0**	54.0
RDLR + †	55.3	**61.8**	54.3	45.9	54.3
RDLR + CWW + † (proposed)	**56.3**	**61.8**	**54.8**	**46.0**	**54.7**

For each corpus and feature configuration of the out-of-corpus SER, the best performance of each target corpus is marked in boldface.

## Data Availability

Datasets mentioned in this paper can be downloaded at the following links (accessed on 23 June 2024): CREMA-D https://github.com/CheyneyComputerScience/CREMA-D, IEMOCAP https://sail.usc.edu/iemocap/, MSP-IMPROV https://ecs.utdallas.edu/research/researchlabs/msp-lab/MSP-Improv.html, MSP-PODCAST https://ecs.utdallas.edu/research/researchlabs/msp-lab/MSP-Podcast.html, LibriSpeech https://www.openslr.org/12.
